# L-carnitine Modulates Cognitive Impairment Induced by Doxorubicin and Cyclophosphamide in Rats; Insights to Oxidative Stress, Inflammation, Synaptic Plasticity, Liver/brain, and Kidney/brain Axes

**DOI:** 10.1007/s11481-023-10062-1

**Published:** 2023-05-04

**Authors:** Olivia Fayez Morid, Esther T. Menze, Mariane G. Tadros, Mina Y. George

**Affiliations:** Department of Pharmacology and Toxicology, Faculty of Pharmacy, Ain Shams University, Cairo, 11566 Egypt

**Keywords:** Doxorubicin, Cyclophosphamide, Synaptic plasticity, Oxidative stress, Chemobrain, Inflammation

## Abstract

Chemotherapy-induced cognitive impairment in cancer patients is known as "chemobrain". Doxorubicin and Cyclophosphamide are two chemotherapeutic agents used in combination to treat solid tumors. L-carnitine was reported for its anti-oxidant and anti-inflammatory activities. The goal of the present study was to elucidate the neuroprotective effect of L-carnitine against chemobrain induced by Doxorubicin and Cyclophosphamide in rats. Rats were divided into five groups: Control group; Doxorubicin (4mg/kg, IV) and Cyclophosphamide (40mg/kg, IV)-treated group; two L-carnitine-treated groups (150 and 300mg/kg, ip) with Doxorubicin and Cyclophosphamide; and L-carnitine alone-treated group (300mg/kg). Doxorubicin and Cyclophosphamide induced histopathological changes in rats’ hippocampi and prefrontal cortices, as well as reduced memory as evidenced by behavioural testing. L-carnitine treatment showed opposite effects. In addition, chemotherapy treatment enhanced oxidative stress via reducing catalase and glutathione levels, and inducing lipid peroxidation. By contrast, L-carnitine treatment showed powerful antioxidant effects reversing chemotherapy-induced oxidative damage. Moreover, chemotherapy combination induced inflammation via their effect on nuclear factor kappa B (p65), interleukin-1β, and tumor necrosis factor-α. However, L-carnitine treatment corrected such inflammatory responses. Furthermore, Doxorubicin and Cyclophosphamide reduced synaptic plasticity via hindering expression of brain-derived neurotrophic factor, phosphorylated cyclase response element binding protein, synaptophysin, and postsynaptic density protein 95 whereas protein expression of such synaptic plasticity biomarkers was enhanced by L-carnitine treatment. Finally, acetylcholinesterase activity was found to be enhanced by chemotherapy treatment affecting rats’ memory while L-carnitine treatment reduced acetylcholinesterase activity. L-carnitine also showed hepatoprotective and renal protective effects suggesting liver/brain and kidney/brain axes as possible mechanisms for its neuroprotective effects.

**Graphical Abstract:**

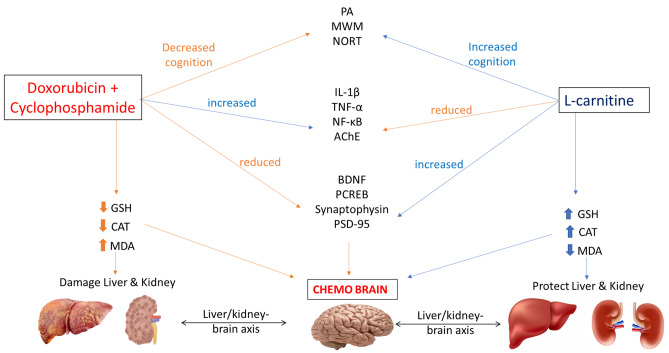

**Supplementary Information:**

The online version contains supplementary material available at 10.1007/s11481-023-10062-1.

## Introduction

Chemotherapy-induced cognitive impairment (CICI), termed “chemobrain” or “chemofog,” refers to the state of extended cognitive impairment resulting from the administration of a variety of chemotherapeutic agents including doxorubicin, cyclophosphamide, 5-fluorouracil, and methotrexate (Christie et al. 2012). It affects nearly 75% of cancer patients during treatment with chemotherapeutic agents (Flanigan et al. 2018; Shi et al. 2019). Exposed cancer patients may suffer difficulty in processing information, inability to perform multi-tasks, difficulties in memory, learning, and attention (Koppelmans et al. 2012). Despite affecting the brain of cancer patients, chemobrain may be also related to reduced kidney and liver function via Qi stagnation (Chao Lu et al. 2018).

Doxorubicin, being an anthracycline, inhibits topoisomerase-II hindering DNA biosynthesis and stopping the process of replication (Rashid et al. 2013). Cyclophosphamide acts by alkylating DNA and the formation of DNA cross-links (Fraiser et al. 1991). Doxorubicin and Cyclophosphamide combination can be commonly used to treat different types of cancers (Kitamura et al. 2017). Doxorubicin was found to undergo redox cycling promoting the release of free radicals and Cyclophosphamide was reported to be transformed by hepatic enzymes producing two metabolites; acrolein and phosphoramide, which could promote free radicals release (Zarei and Shivanandappa 2013; Ibrahim et al. 2021). Such free radicals promote oxidative stress, mitochondrial damage, and hence, cell death.

Although Doxorubicin cannot pass the blood brain barrier (BBB), its effect in the brain may take place through neuroinflammatory mechanisms owing to free radicals production. Such free radicals can pass the BBB and induce the production of inflammatory cytokines (Gazal et al. 2014). Cyclophosphamide was reported to induce oxidative stress upon liver transformation, which in turn, could promote an inflammatory response (Naqvi et al. 2019). Cancer patients on Doxorubicin and Cyclophosphamide treatment were proven to have elevated levels of free radicals leading to overproduction of inflammatory mediators as Interleukin-1β (IL-1β) and tumor necrosis factor-alpha (TNF-α) (Salas-Ramirez et al. 2015). Such inflammatory cytokines release represents the core of the pathogenesis of chemobrain (Shi et al. 2019; Wang et al. 2015).

Synaptic plasticity is the change that occurs to the synapses, both structurally and functionally, affecting learning and memory (Hasan et al. 2011). Cognitive dysfunction was reported to be associated with reduction in synaptic plasticity (Lv et al. 2018). cAMP response element-binding protein (CREB) is a transcription factor that plays a critical role in learning and memory. Once activated by phosphorylation (pCREB), it initiates the transcription of brain derived neurotrophic factor (BDNF) which is responsible for memory formation and synaptic plasticity (Amidfar et al. 2020). Synaptophysin and Postsynaptic density protein-95 (PSD-95) are presynaptic and postsynaptic markers, respectively, which are involved in synaptic plasticity and long term potentiation (LTP) (Liao 2020). The lessened expression of BDNF, pCREB, Synaptophysin, and PSD-95 could reduce synaptic plasticity (Niu et al. 2020). In addition, nuclear factor-κB (NF-κB) activation was found to induce cognitive deficit through hindering the production of BDNF and hence, reducing synaptic plasticity (Wang and Jia 2014).

Oxidative stress not only affects brain, but also affects liver and kidney causing multiple-organ failure. In case of liver dysfunction, toxins may accumulate in the blood and pass to the brain causing hepatic encephalopathy. The latter is associated with cognitive impairment and memory loss (Ding et al. 2020). Also, renal dysfunction was reported to be related to oxidative damage and inflammation resulting in albuminuria. Studies showed that there may be an association between albuminuria and cognitive impairment (Ariton et al. 2021).

L-carnitine is a natural chemical synthesized in human body. It improves the energetic state of the cell by facilitating the uptake of acetyl-CoA into the mitochondria during oxidation process of fatty acids (Zidan et al. 2018). L-carnitine was reported to have antioxidant (Ueno 2015), anti-inflammatory (Ye et al. 2010; Assaf et al. 2012), neuroprotective (Ueno 2015), hepatoprotective (Alshabanah et al. 2010), nephroprotective (Sener et al. 2004), and memory enhancing (Abu Ahmad et al. 2016) properties. Moreover, L-carnitine was reported to have anticancer activity via reducing angiogenesis (Baci et al. 2019).

Therefore, the aim of the present study was to assess the effect of L-carnitine on Doxorubicin and Cyclophosphamide induced chemobrain in rats in addition to study the possible oxidative stress, inflammatory response, synaptic plasticity, liver-brain and kidney-brain related mechanisms.

## Materials and Methods

### Animals

Male Wistar rats with an initial weight of 180–210 g were purchased from Nile Co. for Pharmaceutical and Chemical Industries, Cairo, Egypt. They were housed six per cage under standard experimental conditions; temperature 25 ± 3°C, relative humidity 60%, 12 h light/dark cycle, and food and water *ad libitum*. Rats were acclimatized for two weeks before experimentation.

### Drugs and Chemicals

Doxorubicin was purchased as Doxorubicin^®^, KLAB, India. Cyclophosphamide was purchased as Endoxan^®^, Baxter, India. L-carnitine was purchased from Mepaco, Cairo, Egypt. Anti-β-actin [ab8224], anti-p65 NF-κB [ab16502], anti-BDNF [ab108319], anti-pCREB (phospho S133) [ab32096], anti-synaptophysin [ab32127], and anti-PSD95 [ab238135] were purchased from Abcam, USA. Acetylthiocholine iodide and DTNB were purchased from Merck Sigma Aldrich, USA. All other chemicals and buffers are of the best commercial purity available.

### Experimental Design

Forty rats were randomly divided into five groups (n=8) and treated for three weeks as follows:

Group A (control group) received IV saline once weekly and i.p. saline five times per week for three weeks. Group B received Doxorubicin (4 mg/kg) and Cyclophosphamide (40 mg/kg) IV (rat tail vein) once weekly for three weeks in addition to i.p. saline injection five times per week for three weeks. The doses of Doxorubicin and Cyclophosphamide were selected based on previous research (Salas-Ramirez et al. 2015). Such doses of Doxorubicin and Cyclophosphamide combination are considered typical to human chemotherapeutic doses given IV to maximally mimic real cancer patients. Groups C and D received Doxorubicin and Cyclophosphamide in the same dosage regimen as group B, followed one hour by L-carnitine (150 mg/kg, i.p.) and (300 mg/kg, i.p.), respectively, five times per week for three weeks. Doses of L-carnitine were selected according to previous studies (Rababa’h et al. 2019; Jamali-Raeufy et al. 2021). Group E received IV saline once weekly and L-carnitine at a dose of 300 mg/kg, i.p., five times per week for three weeks.

At the end of the three weeks, behavioural tests were carried out to evaluate cognitive function; Morris water maze, novel object recognition task, locomotor activity, and passive avoidance (n=8). Afterward, rats were sacrificed and blood samples from the abdominal aorta were collected. Then, liver, kidney and brain were excised out from each rat. In addition, hippocampi and prefrontal cortices were dissected out from the brain of the rats. Brain, liver, and kidney samples from different groups (n=2) were fixed in 10% buffered formalin for histopathological examination. Hippocampi and prefrontal cortices samples from all groups were homogenized in potassium phosphate buffer for further biochemical analyses, remaining samples were stored at -80°C in liquid nitrogen for western blot.

### Behavioural Assessment

#### Locomotor Activity

Locomotor activity detector used is equipped with infrared beams with wavelength of 875 nm and scan rate of 160 Hz. Animal movements inside the apparatus caused interruptions to infrared beams that were counted using activity monitor. The locomotor activity was assessed as count per 5 m (Ayoub et al. 2022).

#### Novel Object Recognition Test (NORT)

The apparatus consists of a box with dimensions of 40 x 40 x 60 cm^3^. In the first day, rats were left for 5 m for exploration with the two sides of the box contained two identical objects at the corners. On the second day, one of these objects was replaced by a different new object, and rats were left again to explore the 2 objects. The time taken by the rats to explore the old and new objects was monitored and recorded. Contact time was calculated when the rats began to sniff, look or whisk around the objects (Antunes and Biala 2012).

#### Morris Water Maze (MWM)

The apparatus (Neuroscience, Osaka, Japan) used is a white circular pool filled with water and divided virtually into four equal quadrants. A platform was dipped few millimetres below the surface of the water in one of the quadrants (target quadrant). Three trials were done per day from different release positions (from the three quadrants other than the quadrant that had the platform) for four days. Each trial took 90 s, and the rats that failed to reach the platform within the time allowed were manually guided to it. The fifth day was the probe test day, the platform was removed, and the rats were released from the quadrant opposite to the platform quadrant and were allowed to swim freely for 90 s. Recording system was used to determine the latency of finding the platform. The time spent in the platform quadrant was measured (Gulinello et al. 2009).

#### Passive Avoidance

Passive avoidance apparatus, (UgoBasile, Italy) composed of light and dark compartments with equal sizes separated by an automatic sliding door, was used. On the trial day, rats were placed in the light chamber and after one minute the door was opened. The rats received an electric shock (0.5 mA for 2 s) when they entered the dark chamber with the four paws. A cutoff period of 300 s was employed. After 24 h, rats were placed in the light chamber without delivering an electric shock. The time taken by each rat to step through the dark chamber is called step-through latency (George et al. 2020).

### Histological Examination

Liver, kidney, and brain from different groups were fixed in 10% formalin and then washed with water, and dehydration with serial dilutions of methyl and ethyl alcohol was made. Samples were made clear by xylene and after 24 h, were cut by slide microtome at 4 µm thickness, and were assembled on glass slides. Glass slides were visualized after staining by hematoxylin and eosin using a full HD microscopic camera (Bancroft et al. 2013).

### Serum Markers

#### Creatinine and Blood Urea Nitrogen (BUN)

Serum creatinine and BUN were assessed by kinetic Jaffé and urease and glutamate dehydrogenase-based kinetic reactions, respectively, using kits purchased from Spectrum Diagnostics Co., Cairo, Egypt (Bowers and Wong 1980; Tiffany et al. 1972).

#### Aspartate Aminotransferase (AST) and Alanine Transaminase (ALT)

Liver enzymes, AST and ALT, were determined colorimetrically according to Young et al. (1975); using kits purchased from Spectrum Diagnostics Co., Cairo, Egypt.

### Acetylcholinesterase (AChE) Activity

The activity of AChE was detected according to Ellman et al (1961). Briefly, hippocampi and prefrontal cortices homogenates were prepared in sodium phosphate buffer to release the membrane-bound enzyme. The substrate acetylthiocholine iodide is then added to produce thiocholine which in the presence of oxidizing agent 5,5’-dithio-bis-2-nitrobenzoic acid (DTNB) generates a yellow colour, which was measured at 405 nm. Results were expressed as nM/min/mg tissue.

### Assessment of Oxidative Stress Markers

Catalase (CAT) and reduced glutathione (GSH) levels were determined colorimetrically using kits obtained from Biodiagnostics, Giza, Egypt, according to Aebi (1984) and Beutler et al (1963), respectively, and results were expressed as unit/mg tissue and nmol GSH/mg tissue, respectively. Malondialdehyde (MDA) levels were also measured according to Satoh (1978) method for the assessment of lipid peroxidation. The results were expressed as nmol MDA/mg tissue.

### TNF-α and Il-1β Determination

The levels of TNF-α and IL-1β were analysed using ELISA assay kits obtained from INOVA laboratories (Catalogue No. In-Ra1344 and In-Ra0668, respectively). Briefly, each kit microplate was pre-coated with a specific monoclonal antibody against either TNF-α or IL-1β. Samples and standards were added, then, secondary antibody, and biotinylated polyclonal antibody. Afterward, peroxidase enzyme substrate was added followed by stop solution. Colour intensity was measured at 450 nm using a microplate reader (ChroMate-4300, FL, USA). The concentrations of TNF-α and IL-1β were expressed as pg/mg tissue.

### BDNF, p-CREB, p65 NF-κB, PSD-95, and Synaptophysin Assay Using Western Blot

Samples from different groups were loaded on SDS-PAGE gel with 4 μg proteins content then transferred to polyvinylidene fluoride (PVDF) membrane and blocked in 5% non-fat dry milk in Tris-buffered saline, 0.1% Tween 20 (TBST). The membrane was incubated at 4°C overnight using primary antibody solutions of BDNF, p-CREB, p65 NF-κB, PSD-95, synaptophysin, and β-actin. β-actin was used as reference loading control for all other assessed markers to normalize protein levels. TBST was used for washing then the diluted secondary antibody solution was added, and left for 1 h incubation. Enhanced chemiluminescent substrate was then used to determine protein bands on the membrane, which was captured by the radiographic film.

### Statistical Analysis

Statistical differences between groups were evaluated using one-way ANOVA followed by Tukey as post-hoc test. Data were presented as mean ± SD, and p < 0.05 is considered statistically significant. All statistical analyses and graphs were performed using GraphPad Prism software version 7 (GraphPad Software, Inc., La Jolla, CA, USA).

## Results

### Behavioural Tests

#### Locomotor Activity

**Fig. 1:**
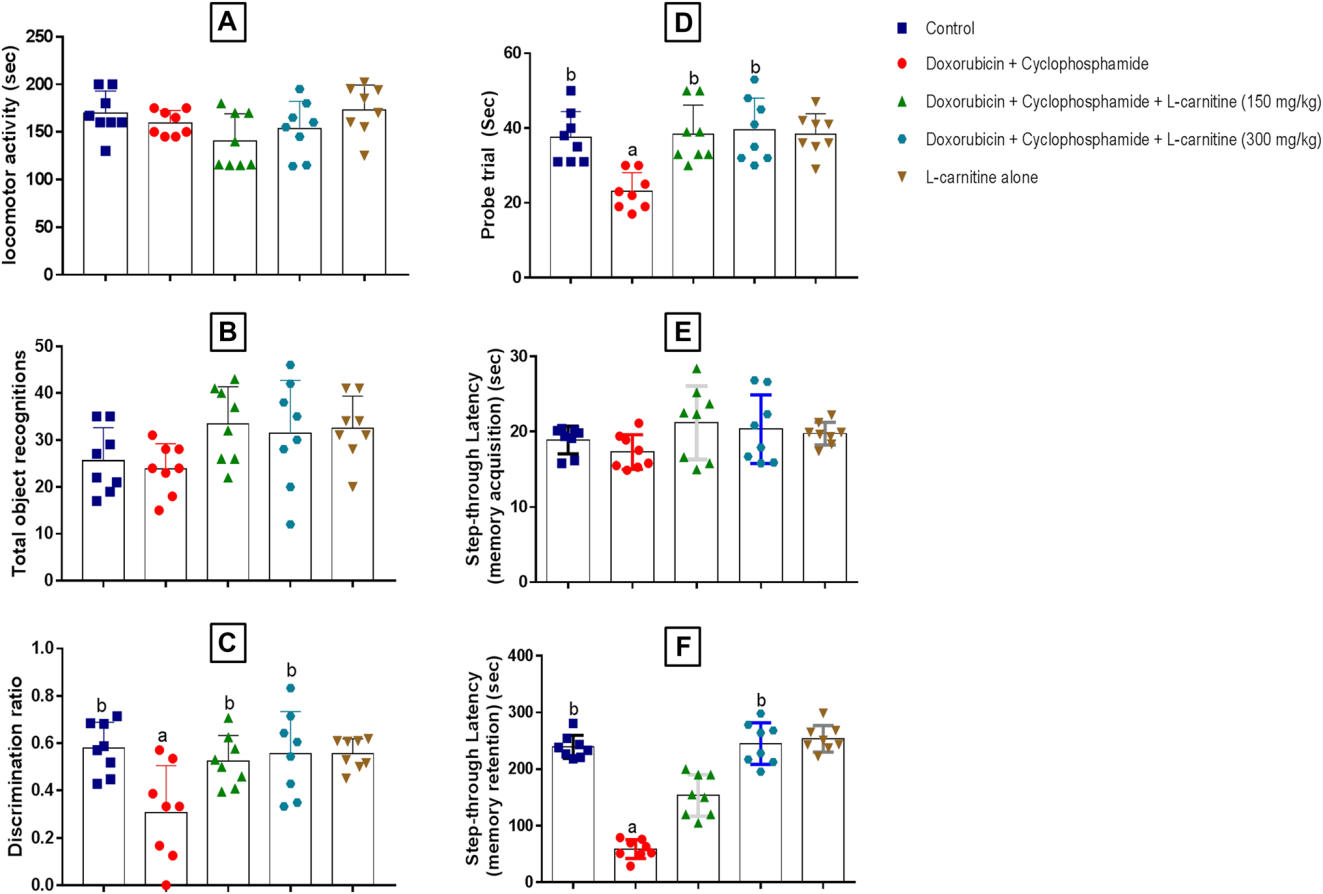
Effect of L-carnitine treatment on locomotor activity (**A**), total object recognition (**B**), discrimination ratio (**C**), probe trial (**D**), memory acquisition (**E**), and memory retention (**F**) against Doxorubicin and Cyclophosphamide-induced chemobrain in rats. Data are presented as mean ± SD (n=8), where: a is statistically significant compared to the control group, b is statistically significant from Doxorubicin and Cyclophosphamide group, c,d are statistically significant compared to the low and high L-carnitine groups, respectively

Insignificant changes were detected for locomotor activity among groups excluding the effect of locomotor activity on other behavioural tests (Fig. 1A).

#### NORT

NORT test was carried out to investigate the effect of L-carnitine on object recognition memory. It was determined in terms of total object recognitions (Fig. 1B) and discrimination ratio (Fig. 1C). There were no significant differences between groups for total object recognitions. However, Doxorubicin and Cyclophosphamide treatment significantly decreased the exploration time of the new object when compared to the control group. On the other side, treatment with L-carnitine, either 150 mg/kg or 300 mg/kg, induced a significant elevation in exploration time as compared to Doxorubicin and Cyclophosphamide-treated group.

#### Spatial Memory (Morris Water Maze) Test

As shown in Fig. 1D, treatment with Doxorubicin and Cyclophosphamide revealed a significant reduction in the probe trial relative to the control group. By contrast, L-carnitine-treated rats, 150 mg/kg and 300 mg/kg presented a significant elevation in the time spent in the target quadrant compared to the chemotherapy-treated group.

#### Memory Acquisition and Memory Retention (Step-Through Passive Avoidance) Test

In the training session (Fig. 1E), there was no significant difference detected among the different treatment groups. Concerning testing day (Fig. 1F), chemotherapy treatment significantly reduced the step-through latency when compared to the control group, whereas high L-carnitine dose treatment statistically increased the time for stepping through the other chamber when compared to Doxorubicin and Cyclophosphamide-treated group.

### Histological Examination

**Fig. 2:**
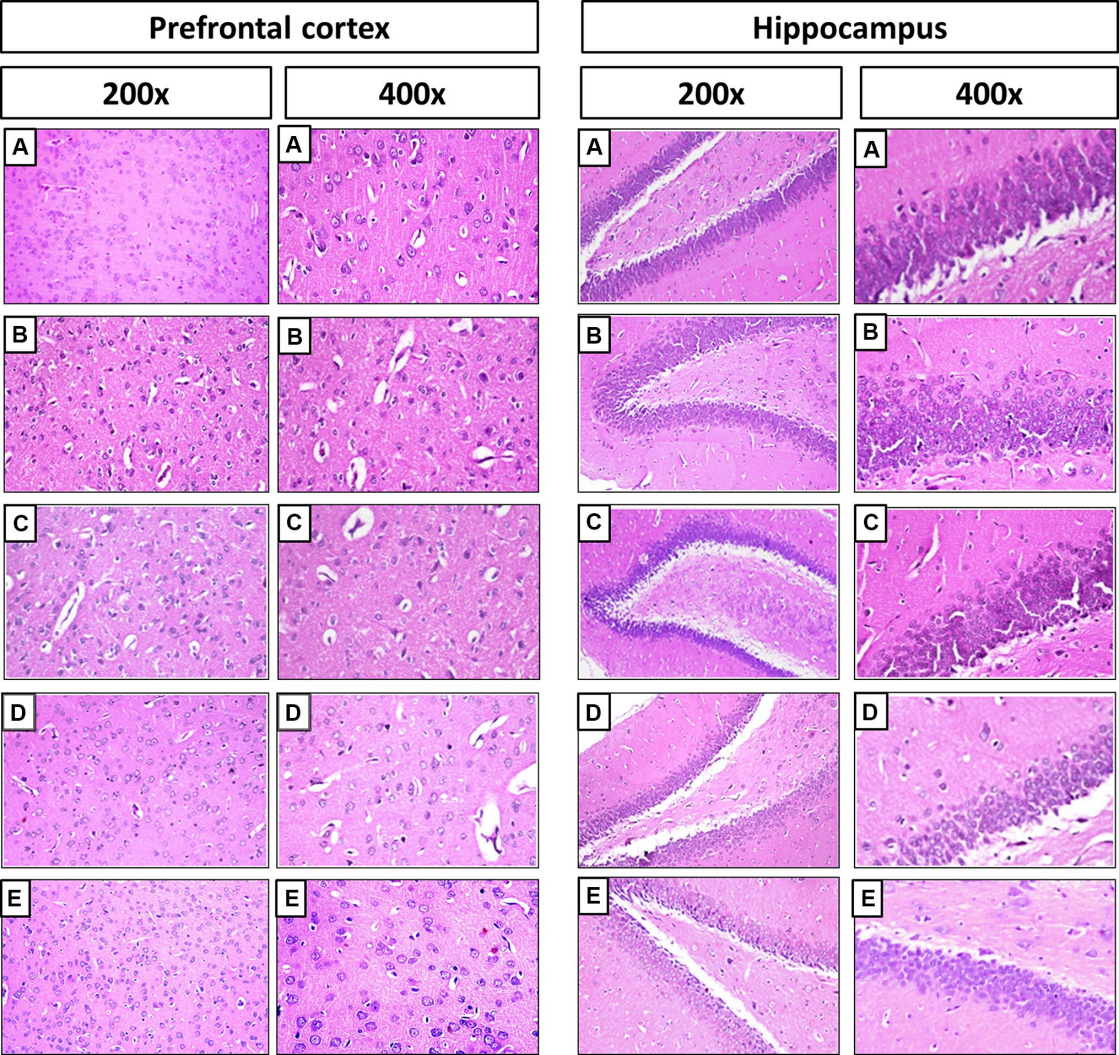
Effects of L-carnitine treatment on Doxorubicin and Cyclophosphamide-induced histological alterations of rats’ prefrontal cortical and hippocampal regions. Photomicrographs of haematoxylin and eosin-stained sections from control group (**A**); Doxorubicin (4 mg/kg) and Cyclophosphamide (40 mg/kg) -treated group (**B**); Doxorubicin and Cyclophosphamide + L-carnitine group (150 mg/kg) (**C**); Doxorubicin and Cyclophosphamide + L-carnitine group (300 mg/kg) (**D**); L-carnitine alone group (300 mg/kg) (**E**)

Regarding the prefrontal cortex, the control group showed normal histological structure with normal neuronal cells (Fig. 2A). However, Doxorubicin and Cyclophosphamide treated rats presented focal gliosis with numerous microglia. In addition, vascular dilatation and perivascular oedema were observed. Neuronal degeneration and apoptosis of neuronal cells were detected (Fig. 2B). Treatment with L-carnitine at a dose of 150 mg/kg revealed mild neuronal degeneration. Gliosis with perivascular oedema was also observed (Fig. 2C). L-carnitine (300 mg/kg)-treated group illustrated fewer numbers of degenerated neuronal cells, as well as mild gliosis and perivascular oedema (Fig. 2D). L-carnitine alone-treated rats presented normal histopathological structures of the prefrontal cortex (Fig. 2E).

For the hippocampus, normal control rats revealed no histopathological alterations in hippocampus areas (Fig. 2A). By contrast, the hippocampal dentate gyrus region of the chemobrain group revealed neuronal disorganization and degeneration. (Fig. 2B). Histopathological examination of rats’ hippocampi treated with L-carnitine (150 mg/kg) showed cellular disorganization (Fig. 2C). Treatment with the higher dose of L-carnitine revealed cellular organization (Fig. 2D). The last group revealed normal layers of hippocampal regions showing three layers of cells, molecular, pyramidal and polymorphic layers with normal structure and arrangement (Fig. 2E). Semi-quantitative scoring of histological changes in hippocampi and prefrontal cortices was performed (Supplementary file S1).

**Fig. 3:**
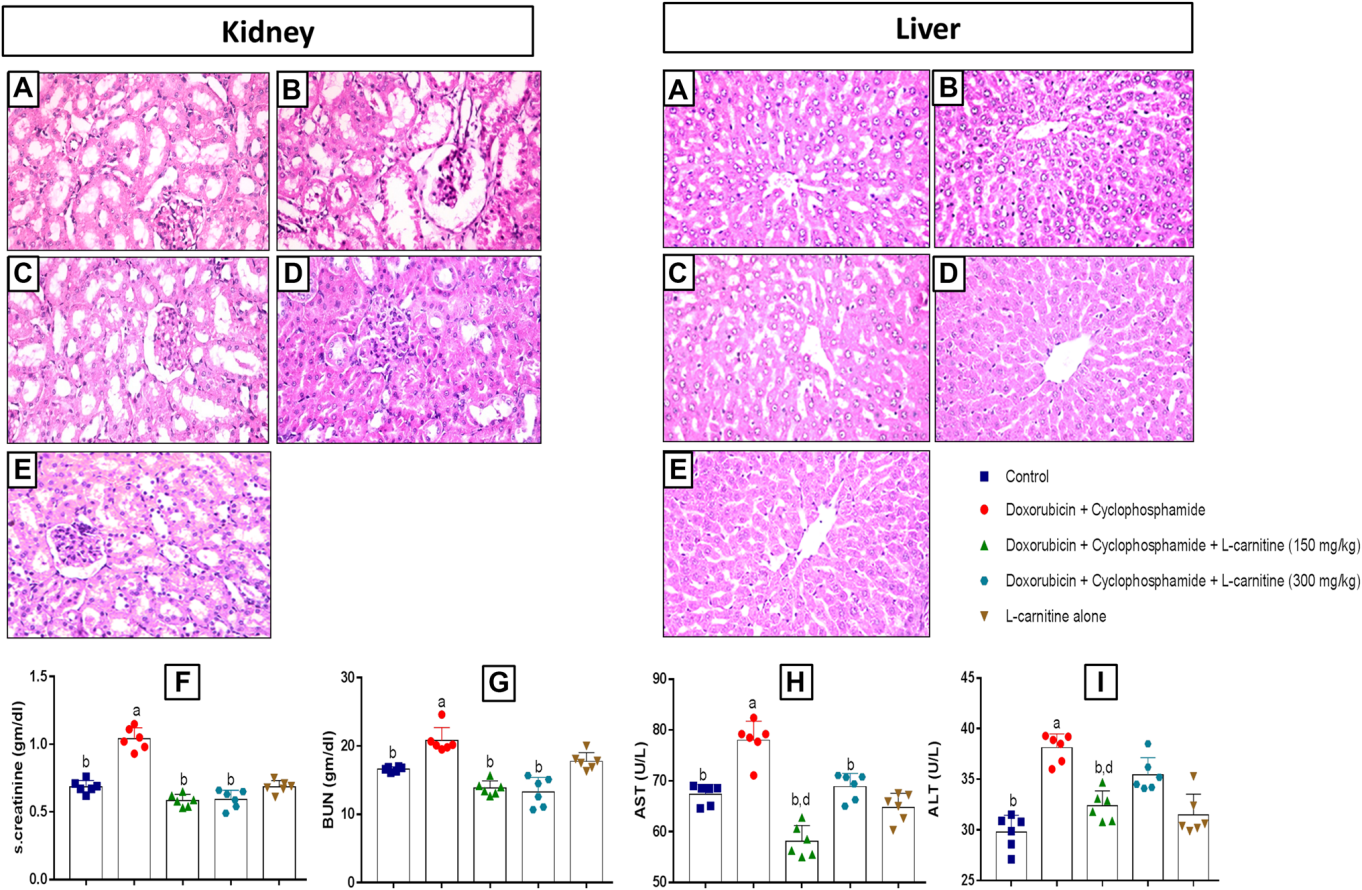
Effects of L-carnitine treatment on Doxorubicin and Cyclophosphamide-induced histopathological alterations of rats’ liver and kidney. Photomicrographs of haematoxylin and eosin-stained sections from control group (**A**); Doxorubicin and Cyclophosphamide-treated group (4/40 mg/kg) (**B**); Doxorubicin and Cyclophosphamide + L-carnitine group (150 mg/kg) (**C**); Doxorubicin and Cyclophosphamide + L-carnitine group (300 mg/kg) (**D**); L-carnitine alone (300 mg/kg) group (**E**); with 400x magnification power. Effect of L-carnitine treatment on Doxorubicin and Cyclophosphamide-induced alteration of serum creatinine (**F**), BUN (**G**), AST (**H**), and ALT (**I**)

For kidney tissues, the control group showed normal histological structure characterized by circumscribed glomeruli with normal structure of capillary tufts and Bowman's capsule (Fig. 3A). By contrast, the chemotherapy-treated group showed shrinkage of capillary tufts with the widening of Bowman's space of some glomeruli. Moreover, degeneration of renal tubular epithelial lining appeared in form of swelling and granularity of its cytoplasm. Tubular epithelial cell necrosis and apoptosis represented nearly 50% of the examined tissue (Fig. 3B). L-carnitine (150 mg/kg) treated group presented hypercellularity of capillary tufts with narrowing of Bowman's space of some glomeruli. Degeneration of renal tubular epithelial lining appeared in form of swelling and granularity of its cytoplasm. Tubular epithelial cell necrosis and apoptosis (nearly 25% of the examined tissue) were observed (Fig. 3C). Higher dose L-carnitine-treated rats revealed normal histological structure of glomerular capillary tufts and Bowman's capsule. The renal tubules of both proximal and distal convoluted tubules showed intact epithelial lining and regular arrangement (Fig. 3D). L-carnitine alone treatment showed normal histological structure of renal tubular epithelial lining which appeared intact with clear lumen without significant necrosis or apoptosis. Circumscribed glomeruli with normal architecture of capillary tufts was also noticed (Fig. 3E).

Concerning liver tissue, the control group showed normal histopathological structure of hepatic lobules and normal organization of hepatic cords with the prominent central hepatic vein (Fig. 3A). Doxorubicin and Cyclophosphamide treated rats revealed disorganization of hepatic cords and necrobiotic changes of hepatocytes. Narrowing of hepatic sinusoids and hyperplasia of Kupffer cells were also noticed (Fig. 3B). Lower L-carnitine dose treatment group revealed ballooning degeneration of hepatocytes, as well as, narrowing of sinusoids with hyperplasia of Kupffer cells. Hepatocytes in the peripheral zone showed mild swelling and narrowing of hepatic sinusoids (Fig. 3C). Rats treated with L-carnitine (300 mg/kg) showed mild swelling of hepatocytes and granularity of its cytoplasm with central situated vesiculated nuclei and peripheral condensation of nuclear chromatin (Fig. 3D). L-carnitine alone-treated group presented normal histological structure of hepatic lobules characterized by normal arrangement of hepatic cords (Fig. 3E). Scoring of hepatic and renal histological changes was recorded in Supplementary file S2.

### Serum Markers

Serum creatinine and BUN were analysed for kidney function assessment. Doxorubicin and Cyclophosphamide-treated group manifested a significant increase in serum creatinine level compared to the control group by 1.51 folds. Low and high L-carnitine doses-treated groups revealed a significant reduction in serum creatinine levels relative to Doxorubicin and Cyclophosphamide-treated group by 1.77 and 1.75 folds, respectively (Fig. 3F). Chemotherapy treatment statistically increased BUN level relative to control group by 1.25 folds. Groups treated with both L-carnitine doses showed a significant reduction of BUN when compared to the chemotherapy-treated group by 1.5 and 1.56 folds for low and high L-carnitine doses, respectively (Fig. 3G).

Serum markers (AST and ALT) were analysed to detect liver functioning. For AST, chemotherapy- treatment significantly enhanced AST levels compared to the control group by 1.15 folds. By contrast, both L-carnitine-treated groups showed a significant decline in AST levels when compared to the disease group by 1.34 folds for low dose L-carnitine (150 mg/kg) and 1.13 folds for high dose L-carnitine (300 mg/kg). Low dose L-carnitine-treated group revealed a significant reduction in AST level as compared to the high L-carnitine dose-treated group by 1.18 folds **(**Fig. 3H**).** For ALT, Doxorubicin and Cyclophosphamide-treatment significantly augmented ALT level relative to control group by 1.27 folds. L-carnitine (150 mg/kg)-treated rats manifested a significant decrease in ALT levels relative to both Doxorubicin and Cyclophosphamide-treated and high L-carnitine dose-treated groups by 1.16 and 1.08 folds, respectively (Fig. 3I).

### AChE Activity

**Fig. 4:**
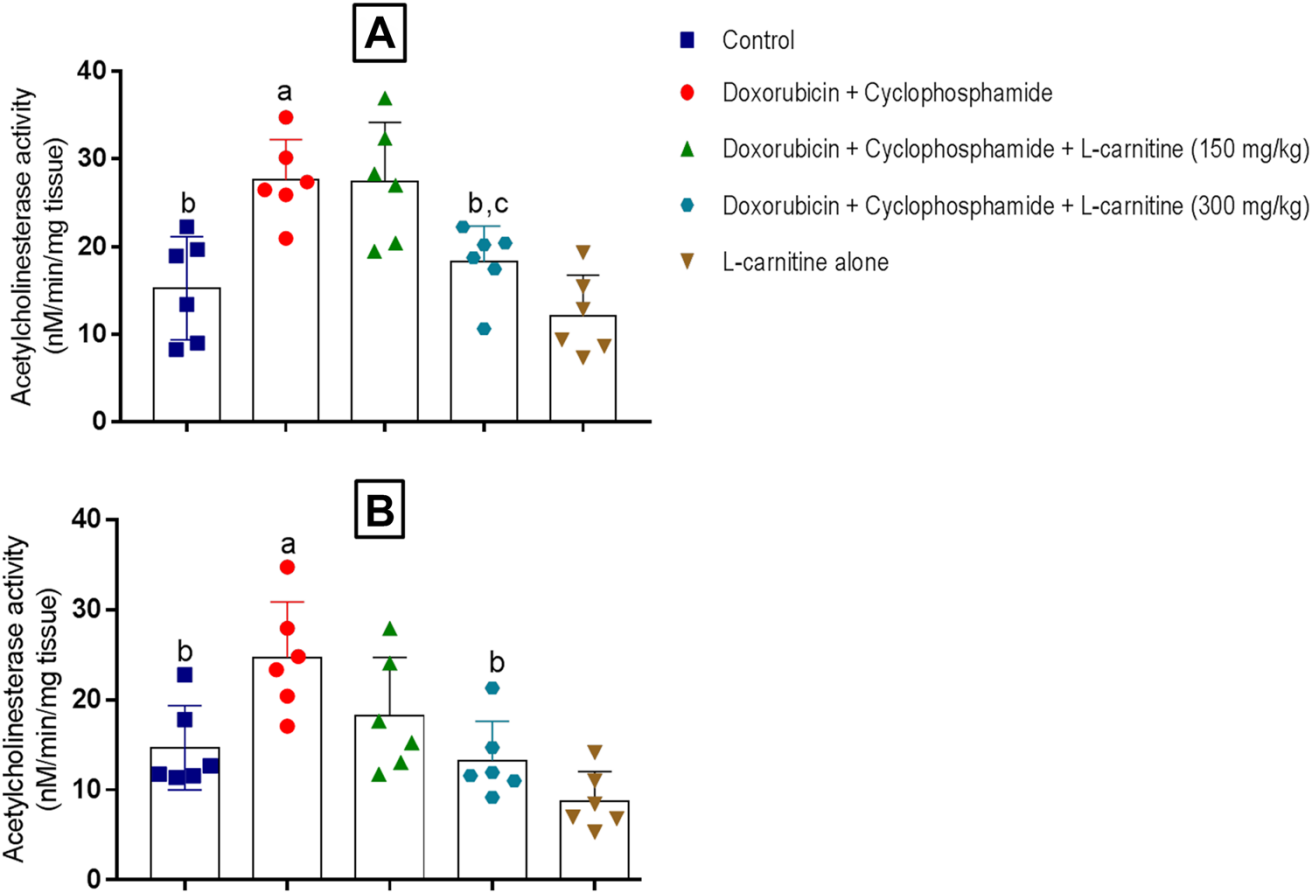
Effect of L-carnitine treatment on Doxorubicin and Cyclophosphamide-induced alteration of prefrontal cortical and hippocampal AChE activity. Data are presented as mean ± SD (n=6) where: a is statistically significant compared to the control group, b is statistically significant from Doxorubicin and Cyclophosphamide group, c,d are statistically significant compared to the low and high L-carnitine groups, respectively, at P<0.05 using one way analysis of variance (ANOVA) followed by Tukey as a post-hoc test

In the prefrontal cortex, Doxorubicin and Cyclophosphamide treatment showed a significant increase in AChE activity compared to the control group by 1.8 folds. However, L-carnitine (300 mg/kg)-treatment statistically decreased AChE activity by 1.5 folds relative to Doxorubicin and Cyclophosphamide-treated group. In addition, L-carnitine (300 mg/kg)-treated rats demonstrated a significant reduction in AChE activity when compared to L-carnitine (150 mg/kg)-treated group by 1.49 folds (Fig. 4A). Regarding hippocampus, chemotherapy treatment significantly increased AChE activity when compared to control group by 1.68 folds. L-carnitine (300 mg/kg)-treated group illustrated a significant decrease in AChE activity relative to chemotherapy-treated group by 1.85 folds (Fig. 4B).

### Oxidative Stress Markers

**Fig. 5:**
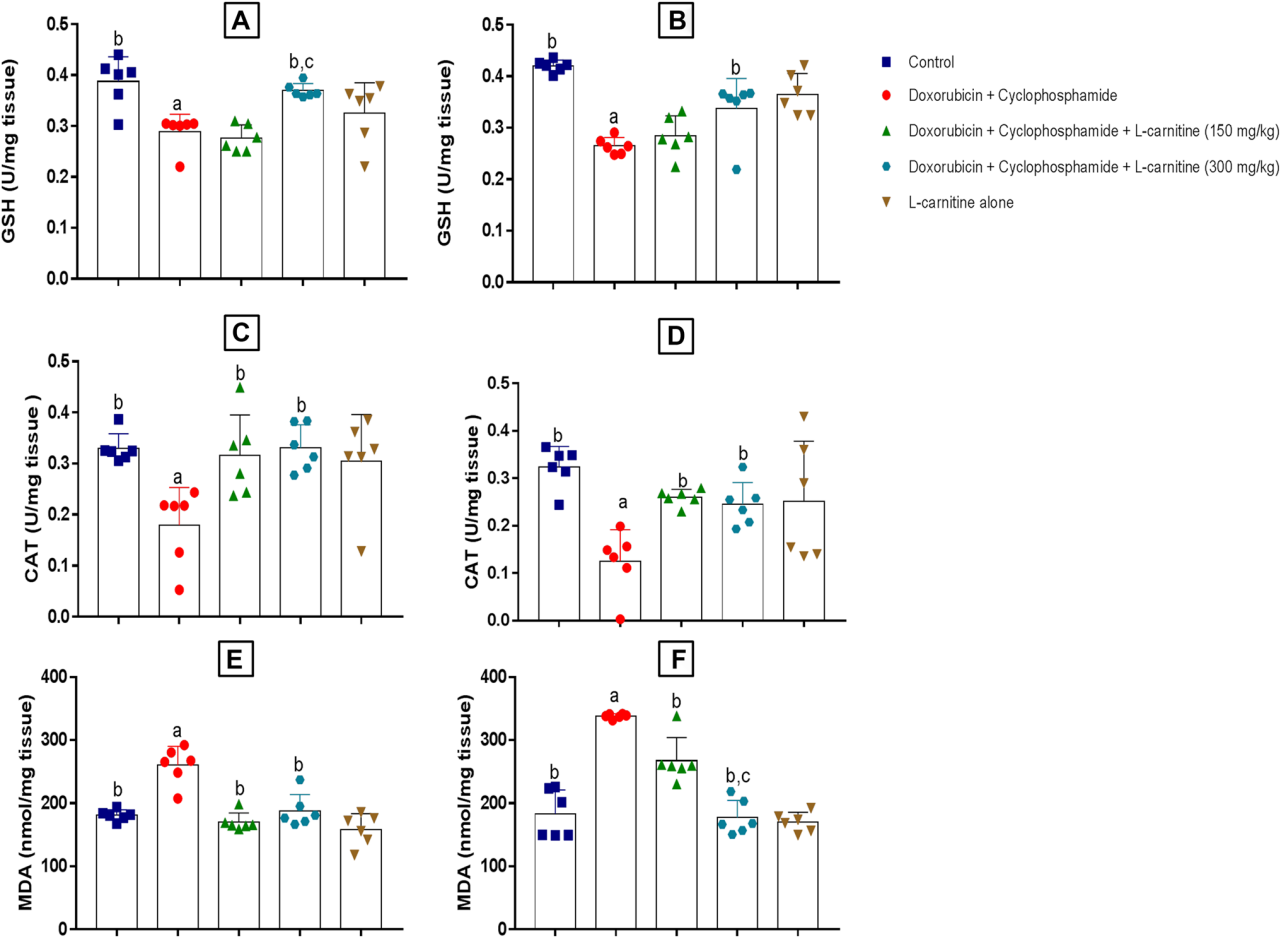
Effects of L-carnitine on prefrontal cortical and hippocampal reduced glutathione (**A**) and (**B**), catalase (**C**) and (**D**), Malondialdehyde (**E**) and (**F**) in Doxorubicin and Cyclophosphamide-induced chemobrain in rats. Data are presented as mean ± SD (n=6) where: a is statistically significant compared to the control group, b is statistically significant from Doxorubicin and Cyclophosphamide group, c,d are statistically significant compared to low and high L-carnitine groups, respectively

The effect of L-carnitine on the level of antioxidant markers such as GSH, catalase, and lipid peroxidation, assessed through measuring MDA levels, was examined in the prefrontal cortex and hippocampus regions of rats (Fig. 5). Concerning the prefrontal cortex, Doxorubicin and Cyclophosphamide significantly ameliorated GSH level compared to control group by 1.34 folds. On the other side, L-carnitine (300 mg/kg)-treated rats showed a significant enhancement in GSH levels compared to Doxorubicin and Cyclophosphamide-treated and L-carnitine (150 mg/kg)-treated groups by 1.27 and 1.33 folds, respectively (Fig. 5A). In the hippocampus, chemotherapy treatment significantly reduced GSH level when compared to control group by 1.58 folds. By contrast, the high L-carnitine dose treated-group showed significant elevation in GSH level compared to the disease group by 1.27 folds (Fig. 5B).

Moreover, Doxorubicin and Cyclophosphamide significantly decreased prefrontal cortical CAT levels compared to the control group by 1.83 folds. Both low and high L-carnitine doses significantly elevated CAT levels compared to the disease group by 1.76 and 1.84 folds, respectively (Fig. 5C). In the hippocampus, Doxorubicin and Cyclophosphamide treatment significantly reduced CAT levels compared to the control group by 2.58 folds. Low and high doses of L-carnitine treatment significantly increased CAT levels compared to the disease-treated group by 2.07 and 1.96 folds, respectively (Fig. 5D).

Concerning MDA, a significant elevation of prefrontal cortical MDA level was observed in Doxorubicin and Cyclophosphamide-treated group relative to the control group by 1.43 folds. On the other hand, L-carnitine treatment, either 150 mg/kg or 300 mg/kg, revealed a significant reduction in MDA level compared to Doxorubicin and Cyclophosphamide-treated group by 1.52 and 1.38 folds, respectively (Fig. 5E). In the hippocampus, Doxorubicin and Cyclophosphamide treatment showed significant elevation of MDA level relative to control group by 1.84 folds. Treatment with both low and high L-carnitine doses statistically reduced MDA levels compared to the chemotherapy-treated group by 1.26 and 2.88 folds, respectively. L-carnitine (300 mg/kg)-treated group statistically hindered MDA level compared to L-carnitine (150 mg/kg)-treated rats by 1.5 folds (Fig. 5F).

### Inflammatory Markers

The effect of Doxorubicin and Cyclophosphamide and L-carnitine on inflammatory markers including IL-1β and TNF-α levels as well as p65 NF-κB expression was assessed in the prefrontal cortex and hippocampus of the treated rats.

#### NF-κB (p65) Protein Expression

**Fig. 6:**
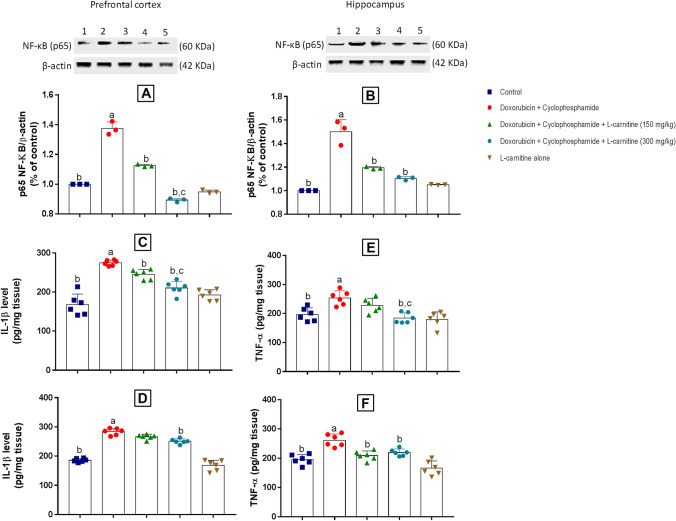
Western blot analysis of prefrontal cortical and hippocampal p65 NF-κB (**A**) and (**B**), and Spectrometric analysis of L-carnitine effect on prefrontal cortical and hippocampal IL-1β (**C**) and (**D**), TNF-α (**E**) and (**F**) in Doxorubicin and Cyclophosphamide-induced chemobrain in rats. Where a is statistically significant compared to the control group, b is statistically significant from Doxorubicin and Cyclophosphamide group, c,d are statistically significant compared to low and high L-carnitine groups, respectively, at P<0.05 using one way analysis of variance (ANOVA) followed by Tukey as a post-hoc test

Regarding prefrontal cortical NF-κB (p65), its protein expression was significantly elevated in Doxorubicin and Cyclophosphamide-treated group as compared to the control group by 1.37 folds. Both groups treated with L-carnitine showed a significant reduction in p65 NF-κB expression when compared to Doxorubicin and Cyclophosphamide group by 1.22 folds for the low dose and 1.53 folds for the high dose. High L-carnitine dose presented a significant decrease in p65 NF-κB expression compared to the low L-carnitine dose by 1.25 folds **(**Fig. 6A**).** In the hippocampus, Doxorubicin and Cyclophosphamide treatment statistically elevated p65 NF-κB expression when compared to the control group by 1.49 folds. However, both low and high doses of L-carnitine treatment induced statistically significant reduction as compared to Doxorubicin and Cyclophosphamide-treated group by 1.25 and 1.35 folds, respectively (Fig. 6B).

#### IL-1β and TNF-α Levels

In the prefrontal cortex, Doxorubicin and Cyclophosphamide treatment significantly elevated IL-1β levels when compared to the control group by 1.63 folds. By contrast, low and high dose L-carnitine-treated groups significantly reduced IL-1β level relative to the disease group by 1.12 and 1.3 folds, respectively. High dose showed significant amelioration in IL-1β level relative to low L-carnitine dose treatment by 1.16 folds (Fig. 6C). Regarding hippocampal IL-1β level, the chemotherapy-treated group revealed a significant increase in IL-1β level relative to the control group by 1.52 folds. By contrast, the group of rats treated with 300 mg/kg L-carnitine presented a significant reduction in IL-1β level relative to chemotherapy-treated by 1.13 folds (Fig. 6D).

Concerning prefrontal cortical TNF-α, Doxorubicin and Cyclophosphamide treatment revealed a significant elevation in TNF-α level as compared to the control group by 1.29 folds. High L-carnitine dose treatment significantly decreased TNF-α level when compared to Doxorubicin and Cyclophosphamide and low dose L-carnitine-treated groups by 1.37 and 1.23 folds, respectively (Fig. 6E). In the hippocampus, 150 and 300 mg/kg L-carnitine treatment significantly decreased TNF-α level as compared to Doxorubicin and Cyclophosphamide-treated group by 1.25 and 1.19 folds, respectively. Doxorubicin and Cyclophosphamide-treated group significantly increased TNF-α levels when compared to the control group by 1.33 folds (Fig. 6F).

### Synaptic Plasticity Markers

**Fig. 7:**
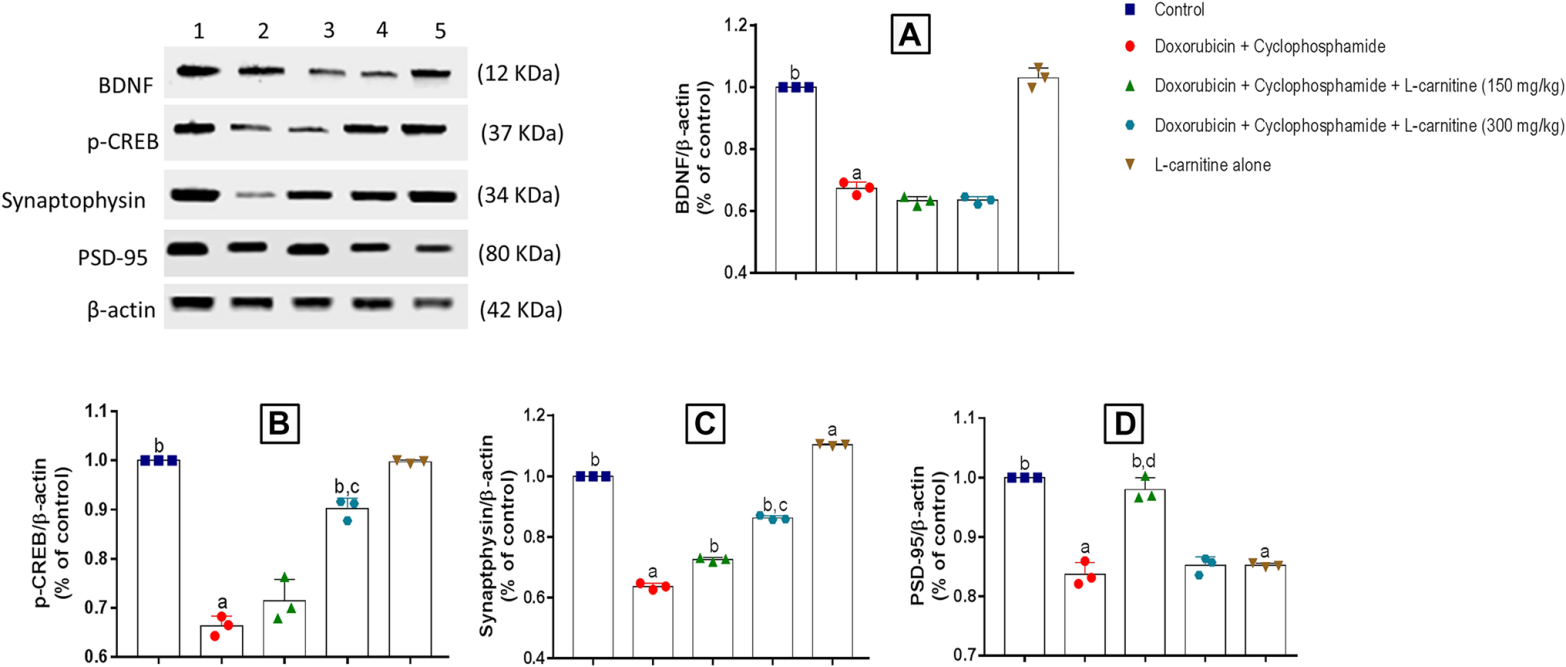
Western blot analysis of prefrontal cortical BDNF (**A**), p-CREB (**B**), synaptophysin (**C**), PSD-95 (**D**) in Doxorubicin and Cyclophosphamide-induced chemobrain in rats. control group (1); Doxorubicin and Cyclophosphamide-treated group (4/40 mg/kg) (2); Doxorubicin and Cyclophosphamide + low dose L-carnitine group (150 mg/kg) (3); Doxorubicin and Cyclophosphamide + high dose L-carnitine group (300 mg/kg) (4); L-carnitine alone group (300 mg/kg) (5). Data are presented as means ± S.D. (n=3) and analyzed by one-way ANOVA followed by Tukey post hoc test. a is statistically significant compared to the control group, b is statistically significant from Doxorubicin and Cyclophosphamide group, c,d are statistically significant compared to the low and high L-carnitine groups, respectively

**Fig. 8:**
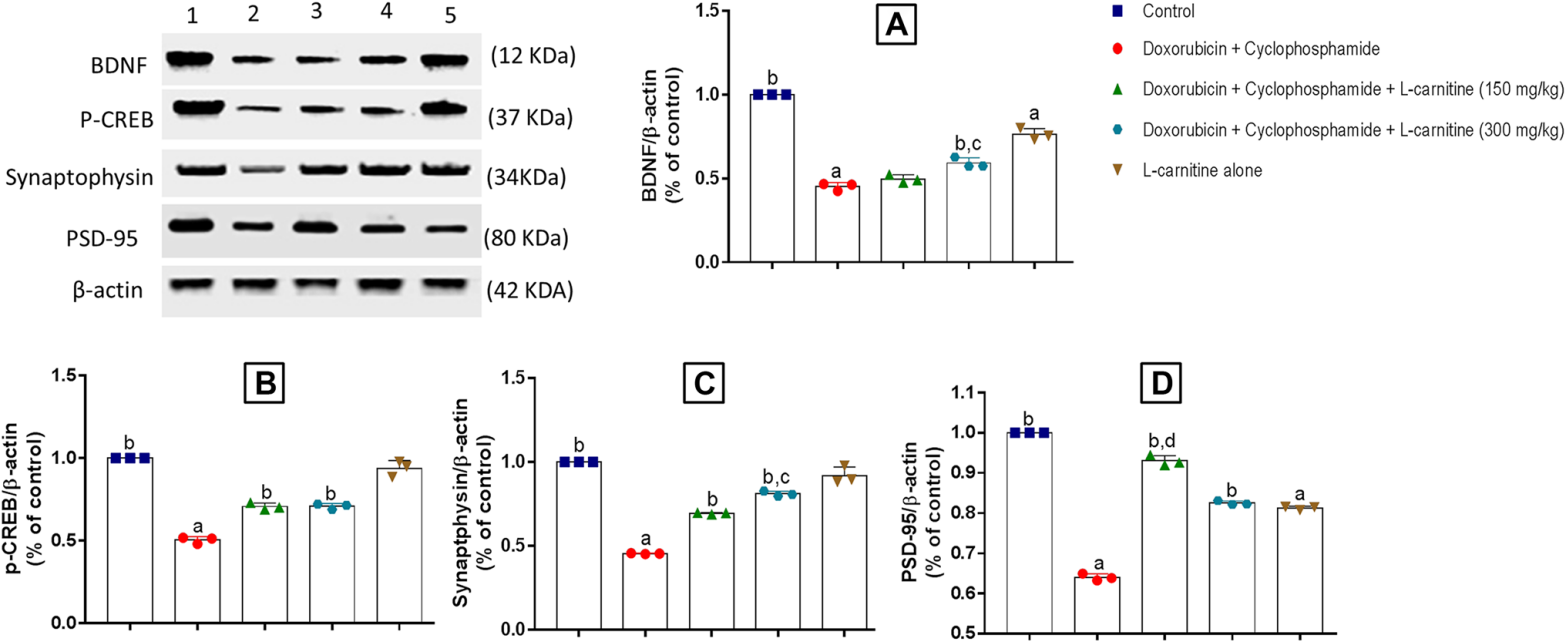
Western blot analysis of hippocampal BDNF (**A**), p-CREB (**B**), synaptophysin (**C**), PSD-95 (**D**) in Doxorubicin and Cyclophosphamide-induced chemobrain in rats. control group (1); Doxorubicin and Cyclophosphamide-treated group (4/40 mg/kg) (2); Doxorubicin and Cyclophosphamide + low dose L-carnitine group (150 mg/kg) (3); Doxorubicin and Cyclophosphamide + high dose L-carnitine group (300 mg/kg) (4); L-carnitine alone group (300 mg/kg) (5). Data are presented as means ± S.D. (n=3) and analyzed by one-way ANOVA followed by Tukey post hoc test. a is statistically significant compared to the control group, b is statistically significant from Doxorubicin and Cyclophosphamide group, c,d are statistically significant compared to the low and high L-carnitine groups, respectively

To detect whether Doxorubicin and Cyclophosphamide treatment could affect synaptic plasticity markers, both presynaptic and postsynaptic markers were measured by western blot analysis (Figs. 7 and 8). BDNF (presynaptic), p-CREB (presynaptic), synaptophysin (presynaptic), and PSD-95 (postsynaptic) proteins were assessed.

In the prefrontal cortex, Doxorubicin and Cyclophosphamide treatment caused a significant decrease in BDNF expression when compared to the control group by 1.54 folds. Both 150 mg/kg and 300 mg/kg L-carnitine-treated groups presented a significant elevation in BDNF expression as compared to Doxorubicin and Cyclophosphamide-treated group by 1.16 and 1.29 folds, respectively. The high L-carnitine dose showed a significant increase in BDNF expression than the low L-carnitine dose by 1.11 folds (Fig. 7A). Regarding p-CREB expression, Doxorubicin and Cyclophosphamide treatment significantly reduced p-CREB expression when compared to the control group by 1.6 folds. Both L-carnitine-treated groups illustrated a significant increase in p-CREB expression as compared to the chemotherapy-treated group by 1.14 and 1.28 folds, for 150 mg/kg and 300 mg/kg L-carnitine, respectively (Fig. 7B).

In addition, synaptophysin was significantly ameliorated by Doxorubicin and Cyclophosphamide treatment relative to control group by 1.56 folds. High L-carnitine dose significantly enhanced synaptophysin expression as compared to Doxorubicin and Cyclophosphamide- and low L-carnitine dose-treated group by 1.35 and 1.18 folds, respectively. Also, the low L-carnitine dose showed significant elevation in synaptophysin expression compared to group B by 1.13 folds. L-carnitine alone group manifested a significant increase in synaptophysin expression relative to control group by 1.1 folds **(**Fig. 7C**).** Furthermore, Doxorubicin and Cyclophosphamide significantly decreased PSD-95 levels relative to the control group by 1.29 folds. Low and high L-carnitine dose-treated groups revealed a significant elevation in PSD-95 expression compared to Doxorubicin and Cyclophosphamide-treated group by 1.15 and 1.2 folds, respectively. Group E presented a significant alteration in PSD-95 expression relative to group A by 1.15 folds (Fig. 7D).

Concerning the hippocampus, the chemotherapy-treated group significantly decreased BDNF expression relative to the control group by 2.21 folds. 300 mg/kg L-carnitine treated-group demonstrated a significant increase in BDNF expression as compared to disease and 150 mg/kg L-carnitine-treated groups by 1.3 and 1.19 folds, respectively. Group E manifested a significant reduction in BDNF expression in comparison to group A by 1.31 folds (Fig. 8A). For p-CREB, Doxorubicin and Cyclophosphamide treatment induced a significant decrease compared to the control group by 1.98 folds. Both low and high doses of L-carnitine statistically elevated p-CREB expression in a similar manner as compared to the chemotherapy treatment by 1.4 folds. L-carnitine alone-treated group manifested a significant change in p-CREB expression relative to the control group by 1.14 folds (Fig. 8B).

Moreover, Doxorubicin and Cyclophosphamide significantly opposed synaptophysin expression compared to the control group by 2.2 folds. Both doses of L-carnitine markedly enhanced synaptophysin expression relative to Doxorubicin and Cyclophosphamide-treated group by 1.52 and 1.78 folds, for the low and the high doses, respectively. However, the high dose of L-carnitine treatment induced a significant increase in synaptophysin expression relative to the lower dose treatment by 1.16 folds. Group E showed significance relative to the control group by 1.12 folds (Fig. 8C). In addition, Doxorubicin and Cyclophosphamide group showed a significant decrease in PSD-95 expression compared to the control group by 1.56 folds. Treatment with either the low or high dose of L-carnitine significantly elevated PSD-95 expression levels compared to Doxorubicin and Cyclophosphamide-treated group by 1.45 and 1.28 folds, respectively. Surprisingly, low L-carnitine dose demonstrated a significant elevation in PSD-95 expression when compared to the high L-carnitine dose-treated group by 1.12 folds. L-carnitine alone-treated group showed a significant decrease in PSD-95 expression compared to the control group by 1.23 folds (Fig. 8D).

## Discussion

Chemobrain is a term recently used to describe the mental fog induced by chemotherapy in cancer patients during or after the treatment course (Shi et al. 2019). Among chemotherapeutic agents used, Doxorubicin and Cyclophosphamide are widely used in combination for solid tumor management (Christie et al. 2012). However, cancer patients on such combination course suffer difficulties in learning and memory activities which could affect their quality of life (Koppelmans et al. 2012). L-carnitine, a natural endogenous product, was reported for several biological activities. The present study aimed at evaluating the neuroprotective effect of L-carnitine against cognitive impairment induced by Doxorubicin and Cyclophosphamide in rats. In addition, the current research sheds the light on the roles of oxidative stress, inflammation, and synaptic plasticity in chemobrain induced by Doxorubicin and Cyclophosphamide. It also showed the deleterious effects of intravenous administration of Doxorubicin and Cyclophosphamide against liver and kidney tissues assuming their possible role in cognitive impairment.

In the present study, treatment with Doxorubicin and Cyclophosphamide combination induced learning and memory impairment evidenced by behavioural assessments; locomotor activity, Morris water maze, NORT, and passive avoidance. Such tests showed the negative impact of Doxorubicin and Cyclophosphamide on spatial memory, short-term memory, novel object recognition, and learning. Such results were in accordance with Kitamura et al. (2017). On the other hand, L-carnitine reversed such deleterious effects. L-carnitine treatment was able to enhance spatial memory in Morris water maze testing, short-term memory in passive avoidance, and new object exploration in the NORT test. It is worthy to mention that the higher dose of L-carnitine displayed better results concerning the passive avoidance test than the lower dose. Such effects of L-carnitine were reported in a valproic acid-induced memory impairment model by Nouri et al (2022).

Such behavioural results were further confirmed by histological examination. Doxorubicin and Cyclophosphamide treatment induced microglial activation, neuronophagia, and apoptosis in the prefrontal cortex whereas the hippocampal dentate gyrus region revealed neuronal disorganization and degeneration. By contrast, such histopathological alterations were reduced by L-carnitine treatment, especially with the higher L-carnitine dose (300 mg/kg).

Since ACh was reported to be the main neurotransmitter involved in learning and memory, it was assessed through the measurement of the AChE activity. AChE is the cholinergic enzyme that hydrolyzes ACh centrally and peripherally. In addition, altered ACh levels could affect synaptic plasticity (Freitas et al. 2006). Increased AChE activity has been reported in the Doxorubicin and Cyclophosphamide-treated group, while L-carnitine treatment ameliorated its activity in both hippocampus and prefrontal cortex. Previous reports showed the effect of Doxorubicin and Cyclophosphamide treatment on AChE activity (Akomolafe et al. 2020; Ibrahim et al. 2021). Interestingly, the higher dose of L-carnitine (300 mg/kg) showed superior effects compared to the lower dose of L-carnitine (150 mg/kg) in the prefrontal cortex region and in the hippocampus. Such results may provide evidence concerning L-carnitine beneficial effects on cognition and memory.

Oxidative stress was reported to play a vital role in CICI, it occurs due to chemotherapy-induced increased levels of free radicles; reactive oxygen species (ROS), and reactive nitrogen species. Also, decreased antioxidant capacity was reported to be associated with chemotherapy treatment (Rummel et al. 2021). Evidence showed that the brain is the highest susceptible organ to oxidative damage. Oxidative stress impairs LTP along with spatial memory (Abu Ahmad et al. 2016). It causes oxidation of the polyunsaturated fatty acids membrane of brain cells (Samarghandian et al. 2017) and the production of inflammatory cytokines. The production of inflammatory cytokines causes microglial activation which could activate NF-κB leading to more production of cytokines such as IL-1β and TNF-α causing a strong inflammatory response, disrupting neuronal function, and finally, neuronal death. Studies proved that the elevated levels of TNF-α and IL-1β in the prefrontal cortex and hippocampus caused a loss of integrity of cortical neurons and a reduction in hippocampal neuronal activity (Shi et al. 2019).

In the current study, oxidative stress was assessed in terms of GSH, CAT, and MDA. The chemotherapy-treated group was associated with decreased antioxidant capacity of GSH and CAT levels, and increased the lipid peroxidation marker, MDA, in the hippocampus and prefrontal cortex. This was reported previously by Ibrahim et al (2021) and Singh and Kumar (2019). On the other side, L-carnitine treatment reversed such oxidative damage induced by Doxorubicin and Cyclophosphamide. Treatment with L-carnitine at a dose of 300 mg/kg showed more powerful antioxidant effects than the lower dose (150 mg/kg) concerning GSH levels in the prefrontal cortex and hippocampus and hippocampal MDA levels. Previous studies confirmed that L-carnitine could suppress oxidative stress and the production of ROS (Jamali-Raeufy et al. 2021) via the suppression of lipid peroxidation (Ueno et al. 2015) and increasing antioxidant enzyme activities. L-carnitine could suppress oxidative stress, inflammation, and hence apoptosis via reducing CD4+ cells, decreasing microglial activation, and reducing inflammatory cytokines production after crossing BBB (Zidan et al. 2018).

Besides its effect on oxidative stress, chemotherapy treatment induced neuroinflammation in rat brains. Such effect was evidenced by increased expression of p65 NF-κB, as well as enhanced IL-1β and TNF-α levels in both examined brain regions. L-carnitine effectively reversed these effects by ameliorating p65 NF-κB expression and reducing levels of inflammatory cytokines (IL-1β and TNF-α). This could be partially explained by the ability of L-carnitine to prevent NF-κB translocation from cytoplasm to nucleus preventing the production of inflammatory cytokines (Salama and Elgohary 2021). It is worthy to mention that the higher dose of L-carnitine treatment presented more significant anti-inflammatory results regarding prefrontal cortical IL-1β level, TNF-α level, and p65 NF-κB expression relative to lower dose treatment.

BDNF is considered the most widely distributed neurotrophic factor in the brain that functions for neuronal growth and survival, LTP, and synaptic plasticity. BDNF was found to play an essential role in the prefrontal cortex and hippocampus for learning and memory (Amidfar et al. 2020). Studies proved that reduced levels of BDNF are one of the pathogenesis mechanisms underlying neurodegenerative diseases (Bathina and Das 2015). CREB is a protein responsible for short-term memory and long-term memory in the hippocampus. Once activated, CREB is converted to its phosphorylated form (p-CREB). The later activation was found to increase transcription of cortical and hippocampal BDNF and send molecular signals necessary for maintaining neuronal survival. CREB/BDNF pathway is essential for synaptic plasticity and cognitive function. Previous studies showed that CREB/BDNF pathway is responsible for the pathogenesis of cognitive impairment (Amidfar et al. 2020). There was crosstalk reported between p65 NF-κB and CREB/BDNF pathway where p65 NF-κB activation could affect CREB/BDNF pathway via decreasing their expression. This could reduce synaptic plasticity and ameliorate memory functioning (Tang et al. 2022).

In the present study, it was found that Doxorubicin and Cyclophosphamide significantly hindered BDNF and p-CREB expression in both brain regions of chemobrain-induced rats while L-carnitine reversed these actions by decreasing p65 NF-κB nuclear translocation and significantly increasing the expression of BDNF and p-CREB. Treatment with L-carnitine (300 mg/kg) illustrated more powerful effects than the 150 mg/kg L-carnitine dose.

Furthermore, western blot analyses were performed for two more synaptic plasticity markers; Synaptophysin (presynaptic) and PSD-95 (postsynaptic). Both markers were responsible for learning and memory (Haddar et al. 2021). Synaptophysin is a synaptic vesicle protein that is involved in neurotransmission (Tata et al. 2021). Based on previous reports, there is a connection between PSD-95 level and memory impairment (Haddar et al. 2021). Decreased PSD-95 level may cause a reduction in spatial memory and impair synaptic plasticity. In the current study, Doxorubicin and Cyclophosphamide combination treatment significantly lowered synaptophysin and PSD-95 expression in the prefrontal cortex and hippocampus affecting synaptic plasticity and memory of rats. On the other hand, L-carnitine could significantly reverse Doxorubicin and Cyclophosphamide effect on rats and elevate synaptophysin and PSD-95 expressions in the treated rats. For synaptophysin, the high L-carnitine dose was more effective in both regions of the brain, but for PSD-95, L-carnitine lower dose showed more significant results in the hippocampus. These findings confirm that administration of L-carnitine during a chemotherapy regimen could alleviate oxidative stress and p65 NF-κB translocation, thereby enhancing synaptic plasticity which could be one of the possible mechanisms underlying the neuroprotective effects of L-carnitine.

Besides its effects on the prefrontal cortex and hippocampus, L-carnitine activity on liver and kidney tissues was further evaluated. Liver and kidney tissue damages were reported to affect neuronal cells and affect cognition. Doxorubicin and Cyclophosphamide combination was reported before to induce oxidative damage in liver and kidney tissues (Samarghandian et al. 2017). Such oxidative damage could affect brain function disrupting memory and learning. Moreover, abnormal kidney function could cause cognitive impairment (Abu Ahmad et al. 2016). So, tracking liver and kidney damage following Doxorubicin and Cyclophosphamide treatment may be a possible way to prevent CICI. In this context, histological examination and detection of serum levels of AST, ALT, creatinine, and BUN were performed to test the effect of chemotherapy and L-carnitine on liver and kidney tissues. Histopathological examination revealed that Doxorubicin and Cyclophosphamide treatment induced severe liver damage whereas L-carnitine-treated groups were mildly affected reversing the deleterious effects caused by chemotherapy treatment. Moreover, Doxorubicin and Cyclophosphamide treatment was associated with moderately damaged renal tissue. However, L-carnitine-treated rats demonstrated mild renal damage ameliorating the toxic effect of chemotherapy treatment on kidney tissue. Such results were further confirmed by serum markers detection. L-carnitine treatment showed a significant decrease in serum levels of AST, ALT, creatinine, and BUN. This may provide a possible role for liver/brain and kidney/brain axes behind L-carnitine-induced neuroprotection.

## Conclusion

This study revealed, for the first time, the effect of L-carnitine on Doxorubicin and Cyclophosphamide- induced chemobrain in rats. L-carnitine positively affected memory acquisition and retention, spatial memory, short-term memory, and learning. In addition, L-carnitine prevented the histopathological alterations induced by Doxorubicin and Cyclophosphamide treatment. Moreover, via its antioxidant properties, L-carnitine demonstrated neuroprotective effects. In addition, L-carnitine reduced neuroinflammation evidenced by its effect on p65 NF-κB and the underlying cytokines: IL-1β and TNF-α. Furthermore, via modulating synaptic plasticity, L-carnitine could oppose cognitive impairment induced by Doxorubicin and Cyclophosphamide. Also, L-carnitine hepatoprotection and nephroprotection may provide a possible clue for chemobrain prevention in cancer patients on Doxorubicin and Cyclophosphamide treatment. These findings conclude that L-carnitine may be a good candidate for the clinical trials evaluation of chemobrain prevention in cancer patients, particularly as L-carnitine is already approved in the market with no serious side effects.

### Supplementary Information

Below is the link to the electronic supplementary material.

Supplementary file1 (PDF 143 KB)

Supplementary file2 (PDF 147 KB)

## Data Availability

Data supporting the results in this article are available upon reasonable request.
