# Supplementary material for: L-carnitine Modulates Cognitive Impairment Induced by Doxorubicin and Cyclophosphamide in Rats; Insights to Oxidative Stress, Inflammation, Synaptic Plasticity, Liver/brain, and Kidney/brain Axes
Source: J Neuroimmune Pharmacol. 2023 May 4;18(3):310–26. doi: 10.1007/s11481-023-10062-1 (PMC10577097; doi:10.1007/s11481-023-10062-1)
Supplement: Supplementary file 1 — Supplementary file1 (PDF 143 KB) [file 11481_2023_10062_MOESM1_ESM.pdf]

Table 1: Histological changes' scoring for brain tissues among different groups.

Group	Prefrontal cortex	Hippocampus
**Control**	0	0
**Doxorubicin (4 mg/kg) + Cyclophosphamide (40 mg/kg)**	3	2
**Doxorubicin + Cyclophosphamide + L-carnitine (150 mg/kg)**	3	1
**Doxorubicin + Cyclophosphamide + L-carnitine (300 mg/kg)**	1	1
**L-carnitine (300 mg/kg)**	0	0

Effect of L-carnitine on chemobrain induced by Doxorubicin and Cyclophosphamide in rats. Doxorubicin (4 mg/kg) and Cyclophosphamide (40 mg/kg) were administered IV, once weekly for 3 weeks. L-carnitine was administered once daily, 5 days per week for 3 weeks in doses of 150 mg/kg and 300 mg/kg. Scorings of histopathological changes were determined in terms of nuclear pyknosis, degeneration in neurons, focal gliosis, and encephalomalacia where;

0: indicates normal histological structure (no damage).
1: indicates mild damage.
2: indicates moderate damage.
3: indicates severe damage.
