# Supplementary material for: L-carnitine Modulates Cognitive Impairment Induced by Doxorubicin and Cyclophosphamide in Rats; Insights to Oxidative Stress, Inflammation, Synaptic Plasticity, Liver/brain, and Kidney/brain Axes
Source: J Neuroimmune Pharmacol. 2023 May 4;18(3):310–26. doi: 10.1007/s11481-023-10062-1 (PMC10577097; doi:10.1007/s11481-023-10062-1)
Supplement: Supplementary file 2 — Supplementary file2 (PDF 147 KB) [file 11481_2023_10062_MOESM2_ESM.pdf]

Table 2: Histological changes' scoring for liver and kidney tissues among different groups.

Group	Liver	Kidney
**Control**	0	0
**Doxorubicin (4 mg/kg) + Cyclophosphamide (40 mg/kg)**	4	3
**Doxorubicin + Cyclophosphamide + L-carnitine (150 mg/kg)**	1	0
**Doxorubicin + Cyclophosphamide + L-carnitine (300 mg/kg)**	2	2
**L-carnitine (300 mg/kg)**	0	0

Effect of L-carnitine on chemobrain induced by doxorubicin and Cyclophosphamide in rats. Doxorubicin (4 mg/kg) and Cyclophosphamide (40 mg/kg) were administered IV, once weekly for 3 weeks. L-carnitine was administered once daily, 5 days per week for 3 weeks in doses of 150 mg/kg and 300 mg/kg. Scoring of histopathological changes in liver tissues was determined where:

Grade 0: No apparent injury
Grade 1: Swelling of hepatocytes
Grade 2: Ballooning of hepatocytes
Grade 3: Lipid droplets in hepatocytes
Grade 4: Necrosis of hepatocytes

Scoring of histopathological changes in kidney tissues was determined where:

Grade 0: Normal histology
Grade 1: Tubular epithelial cell degeneration, without significant necrosis or apoptosis
Grade 2: Tubular epithelial cell necrosis and apoptosis <25%
Grade 3: Tubular epithelial cell necrosis and apoptosis <50%
Grade 4: Tubular epithelial cell necrosis and apoptosis <75%
Grade 5: Tubular epithelial cell necrosis and apoptosis ≥ 75%
